# Behavioural underpinning of mito-nuclear discordances: insights from fire salamanders

**DOI:** 10.1098/rsos.241571

**Published:** 2024-12-11

**Authors:** Andrea Chiocchio, Erica de Rysky, Claudio Carere, Giuseppe Nascetti, Roberta Bisconti, Daniele Canestrelli

**Affiliations:** ^1^ Dipartimento di Scienze Ecologiche e Biologiche, Università degli Studi della Tuscia, Viterbo, Italy

**Keywords:** animal personality, mito-nuclear discordance, mitochondrial DNA, secondary contact, *Salamandra salamandra*

## Abstract

Mito-nuclear discordances across secondary contact zones have been described in a wide range of organisms. They consist of a spatial mismatch between nuclear and mitochondrial genomes in terms of location and extension of the contact zone between distinct evolutionary lineages. Despite the evolutionary and biogeographic causes of mito-nuclear discordances having been extensively investigated, we still lack a clear understanding of their phenotypic underpinnings. Here, we test the hypothesis that mtDNA variation could be associated with behavioural variation and that such association could contribute to asymmetric mitochondrial introgression across a secondary contact zone. We analysed behavioural variation across the mtDNA secondary contact zone of the fire salamander *Salamandra salamandra* in central Italy, which is displaced 600 km from the nuclear contact zone. We found distinct behavioural profiles in the two mitotypes co-occurring in the contact zone. The introgressed mitotype was associated with a ‘slow-thorough’ dispersal profile, characterized by a less active but more cautious and accurate exploration strategy. This pattern was consistent across life stages and contexts: aquatic larvae and terrestrial juveniles, spontaneous activity and response to novelty. These results support the intriguing hypothesis that personality traits associated with distinct mitotypes could contribute to differential mitochondrial introgression and the formation of biogeographic patterns of mito-nuclear discordance.

## 1. Introduction

Secondary contact zones are geographic regions where spatially isolated evolutionary lineages meet after range expansion [1]. They have long been used as natural systems to investigate eco-evolutionary dynamics between and within species [1]. Indeed, when hybridization between divergent lineages occurs, a broad and dynamic range of spatial patterns of genetic variation can arise, leading to a diversity of hybrid zone structures [2,3]. Within this diversity of possible outcomes, considerable attention has been paid to instances of mito-nuclear discordance across contact zones (see [4] for a review), where geographic location and/or spatial extension of a contact zone between mitochondrial genomes do not match with that of nuclear genomes.

Multiple mechanisms have been proposed to explain patterns of mito-nuclear discordance. Some studies emphasized the role of random processes such as genetic drift and demographic disparities on differential hybrid zone movement [5–7]. Other authors emphasized deterministic processes, such as sex-biased mating/dispersal or differential fecundity among hybridizing lineages [8–10]. Among the latter, some stressed the role of positive selection and adaptive introgression of mitochondrial genotypes [11–14]. Accordingly, massive mitochondrial introgression events would occur if the receiving lineage acquires a fitness advantage from the introgressing mitotype rather than fitness costs by mito-nuclear incompatibility [13,15–17]). However, mito-nuclear mismatch could cause incompatibilities between products of the mitochondrial and nuclear genome, resulting in diminished performance or reproductive success [16,18–25]. In fact, because of the involvement of mitochondria in a wide range of phenotypic traits like respiration, metabolic activities, athletic performances, lifespan, sexually selected traits and behaviour [26–30], the identification of any specific selective advantage associated with cases of mitochondrial introgression remains challenging [19].

Dispersal contributes to shaping the spatial patterns of biodiversity over space and time [31–33]. Given the key role of mitochondria in energy production and allocation, a causal-effect link between mtDNA variation and energetically demanding behaviours, such as those promoting dispersal, in explaining mito-nuclear discordance across secondary contact zones seems plausible [34–36]. Interestingly, experimental evidence suggests that mitochondrial introgression can affect animal behaviour in many ways [27], and that novel mito-nuclear combinations obtained by artificial hybridization can generate more active behavioural profiles [37]. In turn, some personality traits have been shown to contribute to modulating individual dispersal [38–43]. For example, fast dispersal profiles are often characterized by bold, aggressive, risk-taking and exploratory attitudes ([41,44–48] but see [49]). Therefore, introgressed mitotypes might carry phenotypic features adaptively linked to higher dispersal capacity, providing phenotypic foundations explaining cases of massive mtDNA introgression [50,51]. Nevertheless, instances of mito-nuclear discordance in natural populations have been so far investigated mainly by analysing the spatial patterns of genetic variation, while we still lack data to understand their phenotypic foundation.

**Figure 1.**
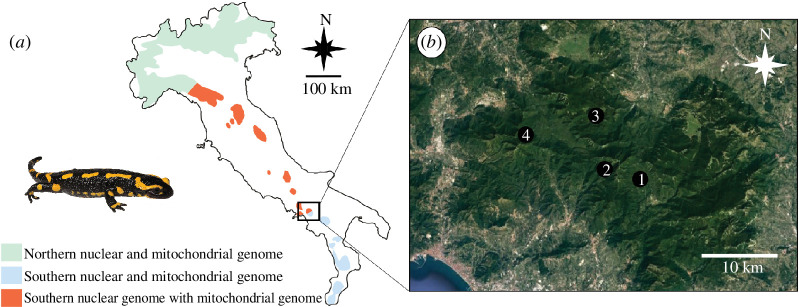
Distribution of the fire salamander *Salamandra salamandra* in the Italian peninsula. (*a*) The study area and (*b*) the Picentini mountain massif (selected area). Photo credit: Giacomo Grignani.

In this study, we investigated the phenotypic underpinnings of a striking case of mito-nuclear discordance in the fire salamander *Salamandra salamandra*. Population genetic studies [52] outlined the occurrence of two distinct evolutionary lineages along the Italian peninsula, *S. s. salamandra* (the northern lineage) and *S. s. gigliolii* (the peninsular lineage [53]), yet unveiled a mito-nuclear discordance in the geographic location of the secondary contact between these lineages. Indeed, while nuclear markers located the contact zone in northwestern Italy, approximately in the Ligurian region, mtDNA markers showed it about 600 km to the south, in south-central Italy (figure 1; [52]). This pattern was explained by a massive southward introgression of the northern mtDNA lineage into the nuclear background of *S. s. giglioli*, following the post-glacial secondary contact among the two lineages in northwestern Italy. Support for this scenario comes from three main lines of evidence (see [52]): (i) the presence of a distinct genetic cluster at nuclear genome for the *S. s. gigliolii* lineage in northern Apennine populations; (ii) a fire salamander fossil record found in the northwestern Apennines, dated back to the last glacial (around 45 000 years BP), which are consistent with a secondary contact zone primarily located in the northwestern Apennines and an mtDNA southward introgression; and (iii) the lack of clines in genetic admixture within the putative area of hybrid zone movement, which undermines the alternative hypothesis of a northward movement of the hybrid zone between nuclear genomes not followed by a movement of the mtDNA contact zone. Notably, the available data suggest that the mtDNA contact zone in south-central Italy occurs in a very short range, and within a fully ‘southern’ nuclear background. Adaptive mitochondrial introgression has been proposed as the most likely mechanism generating such a striking discordance with respect to sex-biased and purely demographic processes [12], particularly in low-dispersal organisms such as salamanders. Thus, the Italian populations of the fire salamander provide an intriguing study system to test the hypothesis of a behavioural contribution to the massive mitochondrial introgression.

To explore this hypothesis, we concurrently characterized mitochondrial, nuclear and behavioural variation within the fire salamander mtDNA contact zone in south-central Italy. In particular, we (i) collected individuals from multiple locations in the surroundings of the mtDNA contact zone identified by Bisconti *et al*. [52] and assessed their mitochondrial genotype (i.e. northern versus southern mitotype); (ii) tested for any geographic pattern of genetic variation at nuclear loci to exclude any effect of local genetic structure on patterns of phenotypic variation; and (iii) scored the salamander inter-individual variation in behavioural traits such as boldness, spontaneous and exploratory activity [41]. We expected to find (i) a link between mtDNA variation and the variation in behavioural traits, irrespective of any spatial pattern of variation at nuclear genetic loci and (ii) personality traits usually linked to higher dispersal tendency (e.g. boldness and exploration attitude [44] associated with the introgressed mitochondrial lineage [50,51]). Furthermore, to investigate putative carry-over effects among distinct life stages, behavioural variation was analysed both in the pre-metamorphic larval stage and in the same individuals at the post-metamorphic life stage, which is the stage mainly responsible for terrestrial movements in this species, since both these stages—and the associated carry-over effects—could be implicated in adaptive and non-adaptive processes leading to mito-nuclear discordance.

## 2. Methods

### 2.1. Study species

The fire salamander *S. salamandra* is an ovoviviparous urodele amphibian (Salamandridae) widespread in temperate and boreal forests of Europe [53]. Females lay aquatic larvae in small brooks and ponds, and larvae metamorphose after 1–3 months; juveniles have a fully terrestrial habit. Sexual maturation occurs after 2–3 years.

Dispersal mainly occurs in larvae and post-metamorphic juveniles: larvae mainly move through passive drift, yet short-range active dispersal (i.e. across ponds close to the natal location) might occur due to resource paucity and/or overcrowding [54]. Metamorphosed juveniles represent the main dispersal stage as individuals emerge from the water bodies and explore the new environment to look for terrestrial food and refugia [55,56]. Conversely, adults show strong breeding site fidelity and territorial behaviour and do not substantially contribute to the dispersal [57–59].

### 2.2. Sampling and housing

We collected 163 larvae across the secondary contact zone between the two mitochondrial lineages in the Picentini mountains, central Italy (see figure 1 and table 1). To reduce the probability of kinship among collected individuals, larvae were collected from different breeding sites located a few kilometres apart. To minimize potential environmental and phenological differences among sampling sites, we selected the sampling sites between 650 and 1100 m.a.s.l. and in similar habitats, i.e. small brooks in beech forests. To collect larvae immediately after their deposition, we monitored the sampling sites weekly since the late winter, and daily after we observed the first larvae at lower altitude sites in the surroundings. The larvae were collected in mid-April, on the same day for localities 1, 2 and 4, and 4 days later for locality 3. Sampled larvae were immediately transported to the housing facilities in small boxes containing stream water (2 l) and kept in dark and fresh containers. Larvae were then individually housed under controlled conditions (water temperature 10–12°C and natural photoperiod). We used plastic baskets (10 × 10 cm) as housing containers, with half a terracotta saucer as shelter, all set in collective PVC tanks filled with dechlorinated soft water. Larvae were fed ad libitum 3 days a week with live *Chironimidae* and *Tubifex*. After metamorphosis, juveniles were individually housed in the same room under controlled conditions (temperature 18–20°C, humidity 65–70% and natural photoperiod). To house juveniles, we used transparent and micro-perforated plastic boxes (11.5 × 11.5 × 6 cm) as terrariums, containing coconut litter, gravel, dechlorinated soft water and a piece of cork as shelter. Juveniles were fed ad libitum with small *Acheta domestica* and *Tenebrio molitor*. All the surviving individuals were released to the collection sites after the experiments.

**Table 1.** Geographic location and sample size of the four sampling locations within the study area.

site code	location	latitude	longitude	altitude (m.a.s.l.)	sample size
1	Montella	40°45'32" N	15°2'16" E	652 m	8 (north: 0; south: 8)
2	Giffoni	40°46'04″ N	14°59'34″ E	750 m	63 (north: 55; south: 8)
3	Piani di Verteglia	40°49'15″ N	14°58'32" E	1170 m	36 (north: 0; south: 36)
4	Serino	40°48'05″ N	14°53'15" E	840 m	56 (north: 26; south: 30)

To obtain individual genotypes, we collected biological tissue for DNA extraction using skin swabs [60]. Since the potential influence (if any) of the skin-swabbing procedure on behavioural tests cannot be predicted, this procedure was carried out at the end of the experiments. That is, the entire set of experimental tests was blind to the mtDNA identity of the studied individuals. Before swabbing, individuals were anaesthetized by submersion in a 0.015 m/v solution of tricaine methane sulfonate (MS222). Then, swabs were stored in ethanol (96% at −20°C) until DNA extraction.

### 2.3. Genotyping

DNA extractions were performed using the standard cetyltrimethylammonium bromide protocol [61]. mtDNA haplotypes were assessed using the polymerase chain reaction coupled with restriction fragment length polymorphism (PCR-RFLP) diagnostic technique. Diagnostic RFLP profiling was selected using the method implemented in the software REPK (Restriction Endonuclease Picker [62]). REPK searches among commercially available restriction endonucleases that uniquely differentiate designated sequence groups. We (i) entered in REPK all the mitochondrial haplotypes from the northern and southern lineages retrieved in Bisconti *et al*. [52], which were differentiated by at least 25 mutational steps; (ii) defined two subgroups (northern and southern haplotypes); and (iii) digested them *in silico* maintaining the default options for defining diagnostic restriction profiles. REPK identified the enzyme *HaeIII* as the best in determining a diagnostic profile in the Cytochrome B (CytB) gene fragment. Then, for all the individuals, a 722 bp portion of CytB was amplified by PCR, following the protocol of Bisconti *et al*. [52], and subsequently digested by *HaeIII* (Promega) according to the manufacturer’s instructions. Restriction fragments were then separated on 1.5% agarose gel and visualized using UV illumination. Ten specimens from Bisconti *et al*. [52] representative of the two lineages were used as reference controls.

To assess the effect of local-scale population genetic structure on behavioural variation and their effect on correlative analyses, all the individuals were genotyped at 13 microsatellite nuclear loci (*SST-A6-I, SST-A6-II, SalE-14, SST-B11, SST-G9, SalE12, SalE2, SST-C3, SalE5, SalE7, Sal3, SalE06, SalE8*), which have proved to be informative at fine geographic scale in this species [63]. In the case where we find the two mitotypes associated with different behavioural profiles, we expect two alternative scenarios of population genetic structure at nuclear loci: (i) population genetic structure overlapping the distribution of the two mitochondrial lineages/behavioural profiles, i.e. the pattern of gene flow could have affected the behavioural differentiation, and (ii) no spatial pattern of genetic structure related to the distribution of the two mitochondrial lineages/behavioural profiles, i.e. we can exclude the effect of patterns of gene flow on behavioural differentiation, supporting an intimate link between mitochondrial and behavioural variation. Laboratory procedures for microsatellite amplification followed previously described protocols [64,65]. Forward primers were fluorescently labelled, and PCR products were electrophoresed by Macrogen Inc. on an ABI 3730xl genetic analyser (Applied Biosystems) with a 500-HD size standard. Microsatellite raw data were analysed using GeneMapper® 4.1. Individuals with more than 50% of missing data were excluded from the subsequent analyses (*n* = 11). Micro-Checker 2.2.3 [66] was used to test for null alleles and large-allele dropout in our data. Allelic frequencies were then computed with GENETIX 4.05 [67], and FSTAT [68] was used to test for deviations from the expected Hardy–Weinberg and linkage equilibria, using the Bonferroni correction for multiple tests.

The genetic structure of populations within the study area was evaluated using the Bayesian clustering method implemented in STRUCTURE [69]. The analysis was carried out using the admixture model without using sampling location as prior information. Preliminary analyses were conducted to assess model performance, with 80 000 steps (the first 20 000 discarded as burn-in) and three replicates for each *K* value (i.e. the number of clusters) between 1 and 4. The final analysis contained 10 replicates for each *K* value, with each run of length 2 00 000 steps, discharging the first 50 000 steps as burn-in. The optimal value of *K* was selected by means of the ln Pr(X|K) method [69] and the Evanno ΔK method [70]

### 2.4. Behavioural assays

**Figure 2.**
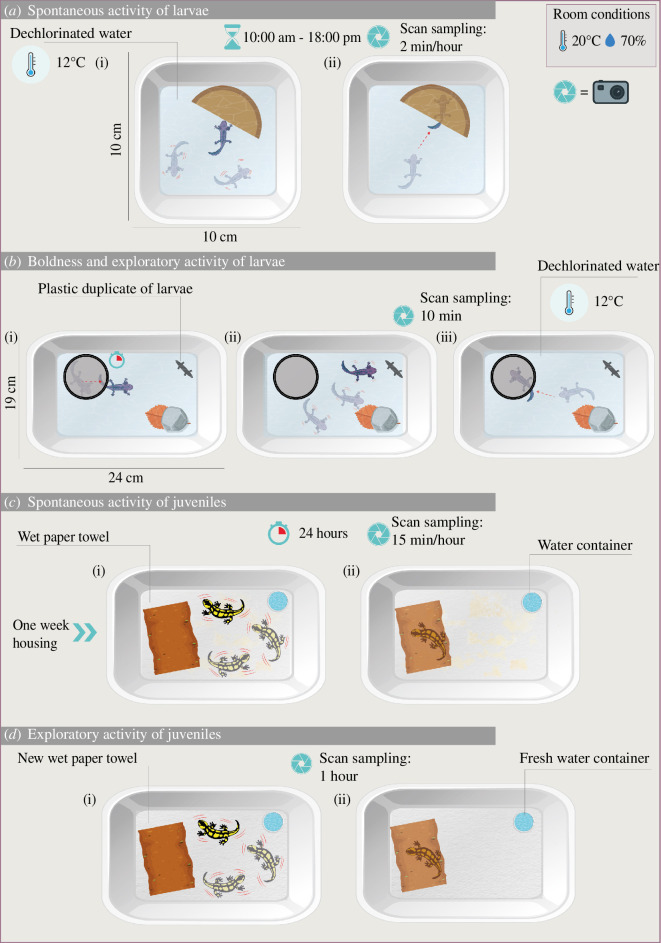
Schematic representation of the behavioural tests conducted on *Salamandra salamandra*. (*a*) Test of the spontaneous activity of larvae in the familiar environment, measuring mobility events (i) and the sheltering behaviour (ii); (*b*) test of boldness and exploratory activity of larvae in a novel environment: boldness was quantified by measuring the latency to exit from the shelter (i), exploration was quantified in terms of time spent in locomotor activity on the total test duration (ii) and the sheltering behaviour was measured as the number of times the larva comes back into the shelter (iii); (*c*) set-up for testing the spontaneous activity of juveniles in a familiar environment, measuring mobility events (i) and the sheltering behaviour (ii); (*d*) set-up for testing the spontaneous activity of juveniles in a novel environment, represented by a new paper towel, shelter and water container, and measuring mobility events (i) and the sheltering behaviour (ii); further details are provided in the main text (graphic design by Eustacchio Montemurno).

The following personality traits were assessed on both larvae and juveniles: spontaneous and exploratory activity, boldness and sheltering behaviour (see electronic supplementary material, table S1 for a summary). Larvae were tested after one week of acclimatization upon arrival; juveniles were tested three months after the metamorphosis. To assess repeatability, each behavioural test was performed twice, after a 7-day interval [71]. All individuals were fasted 48 h before the tests. During the tests room temperature was kept at 20°C and room humidity at 70%. Each behavioural test was recorded using digital cameras (see the next paragraphs for details), and videos were analysed manually with the BORIS software [72] by one of the authors (E.dR.). The tests were set as follows:

- *Spontaneous activity of larvae*. The spontaneous activity in the familiar environment (hereafter FE) was measured in the housing container by scan sampling [73]. We recorded the activity events with GoPro cameras at predetermined time intervals (2 min h^−1^, from 10.00 to 18.00, for a total of 18 min), and counted the number of times the animal made any locomotor activity. We also recorded the sheltering behaviour by counting the number of times the animal was inside the shelter with all four paws in.
- *Boldness and exploratory activity of larvae*. The boldness and the exploration were measured in an unfamiliar, novel environment (hereafter NE), similarly as in other amphibians including our study species (e.g. [74] common frog tadpoles; [75] fire salamander larvae; [76] adult tree frogs). Boldness was quantified by measuring the latency to exit from the shelter, and exploration was quantified as time spent in locomotor activity on the total test duration (exploratory activity percentage on 10 min). NE is assumed to represent a risky situation [73], thus high activity levels and short latencies were associated with an exploratory and bold personality [77]. The sheltering behaviour was recorded as in the FE. An individual was considered immobile if it did not change position for more than 3 s. The test arena consisted of a glass tank (24 × 19 cm) filled with dechlorinated soft water (2 l, 12°C), containing a plastic leaf with a stone on the top. A plastic duplicate of a salamander larva was set at opposite corners of the arena (see figure 2), to possibly also enhance social exploration; however, it was never clearly approached, and we were not able to extract any behavioural descriptor in response to this stimulus. Each larva was gently inserted in a PVC tube as shelter and kept dark for 5 min before the tube opening. Outlier individuals, who had never exited from the shelter or exited immediately (latency < 1 s), were removed from subsequent analyses.
- *Spontaneous activity of juveniles*. The spontaneous activity of juvenile salamanders in the FE was measured in the housing container by scan sampling. Juvenile salamanders did not show high levels of activity; therefore, in order to capture enough movements, we chose to extend the recordings to 24 consecutive hours, taking a picture of the arena every 15 min (for a total of 96 observations) and recording any change of position between each consecutive frame (occurrence of activity events). This measure has proved to be an effective proxy of the total activity level in a subset of individuals where we also scored the whole 24 h recordings. Furthermore, using infrared cameras, we also recorded the sheltering behaviour, as defined above for larvae. To ensure a clear view of the animals, the coconut litter was substituted with a wet paper towel. Animals were kept in this condition for one week before the test.
- *Exploratory activity of juveniles*. The exploration was measured in the NE and was quantified in terms of the percentage of time spent in locomotor activity on the total test duration (exploratory activity percentage on 1 h). An individual was considered not moving at all if it did not change position for more than 3 s. Using infrared cameras, we also recorded the sheltering behaviour, as defined above. The arena set was the same as the FE arena (see above), and, following preliminary observations of juvenile behaviour, the novel situation was considered a change in conditions represented by renewing each paper towel and changing the water container and the shelter before starting the trial.

A graphic representation of each test is shown in figure 2.

### 2.5. Statistical analysis

Individual behavioural consistency was assessed by calculating repeatability estimates on repeated measures for each behavioural descriptor using the mixed models implemented in the ‘rpt.’ function of the ‘rptR’ R package [78,79]. Repeatability coefficients ‘*R*’, that is, intraclass correlation coefficients, were calculated as the ratio of between-individual variance to total variance with linear mixed effects models (LMM) for Gaussian distributed data, using individual identity as a random factor and the link-scale approximation of *R*; activity percentage in the NE for both larvae and juveniles was square root transformed and the normal distribution of residuals was checked by a Kolmogorov–Smirnov test (see [80]). Repeatability coefficients for the latency to exit from the shelter of larvae in NE and for the sheltering behaviour were estimated by fitting generalized mixed effects models for Poisson distributed data, accounting for over-dispersion [79]. The 95% confidence intervals (CI) around repeatability estimates were generated by performing 1000 parametric bootstrap iterations. Variables were considered ‘highly’ repeatable if *R* > 0.5 or ‘marginally’ repeatable if *R* > 0.2 [71,81–83].

The correlation between repeatable behavioural traits, both within and between life stages, was evaluated by estimating the patterns of covariance between pairs of traits running mixed-effects multivariate models [84–86], using the Bayesian approach implemented in the ‘MCMCglmm’ R package [87]. We fitted the models by contrasting each pair of traits in bivariate models, setting the individual ID as a random factor, a non-informative prior (i.e. expected variance *V* = diag (2) and degree of belief ν = 1.002), 1 500 000 iterations thinned each 100 after a burn-in of 500 000 iterations. The fit of the models was evaluated by the visual inspection of the posterior density plots to ensure proper model mixing and convergence. All estimates were presented as posterior median with associated 95% CI (credible interval); phenotypic correlations were considered supported when the credible intervals did not overlap zero.

The effect of mitotype in explaining the inter-individual behavioural differences was tested by running generalized linear mixed effects models (GLMMs) implemented in the ‘lme4’ R package. We considered only repeatable behaviours. Each model was run, entering the behavioural descriptor as the dependent variable, the mitotype as a fixed factor, the trial number as a further fixed factor and the individual ID as a random intercept. A Poisson distribution with log link function was set for the mobility events of larvae and the sheltering of juveniles in FE; a negative binomial distribution was used for the sheltering of juveniles in NE; a Gaussian distribution with identity link function was set for all the other models.

Finally, we tested the effect of mitotype in explaining the behavioural differences across all the repeatable traits of larvae and juveniles together by implementing a permutational multivariate analysis of variance (PERMANOVA) as implemented in the ‘vegan’ R package, which is the non-parametric corresponding multivariate approach to univariate ANOVA, although in this case the significance value is computed by permutations [88]. To do this, we first computed a multivariate trait dissimilarity matrix between each pair of individuals using Euclidean distance based on scaled trait values (i.e. values were standardized using the *decostand* function). To avoid missing data in the dissimilarity matrix, which is not allowed in PERMANOVA next steps, we removed from the dataset the individuals with missing data at more than three traits (32 individuals removed). Then, we used the multivariate trait dissimilarity matrix as a response variable and the mitotype as a predictor in the PERMANOVA in the *adonis2* function of the ‘vegan’ R package; significance was evaluated by setting 999 permutations. Also, the multivariate homogeneity of group dispersions (variances) was evaluated by running the dissimilarity matrix into the *betadisper* function of the same package.

## 3. Results

### 3.1. Genotyping

According to the PCR-RFLP diagnostic profile produced by the restriction enzyme *HaeIII* on the CytB gene fragments, 81 out of 163 individuals showed the northern lineage mitotype, and 82 showed the southern lineage mitotype.

The final microsatellite dataset comprised multi-locus genotypes for 152 individuals at 13 loci. Micro-Checker 2.2.3 detected no traces of null alleles and large-allele dropout in our data. There were no significant deviations from Hardy–Weinberg or linkage equilibria after applying the Bonferroni correction for multiple tests. The number of alleles per locus ranged from 2 (*SST-G9*) to 13 (*SalE2*). The analyses conducted in STRUCTURE clearly showed that *K* = 1 best fit the data, revealing no traces of genetic structure in the study area. Indeed, although both the ln Pr(X|K) and Evanno’s methods addressed 3 as the best clustering option, the inspection of the bar plots for all putative *K* values revealed no biologically meaningful population structures for *K* > 1*,* i.e. each individual is a complete admixture of each estimated cluster (typically occurring when there is complete panmixia (see [89]). Results from the Bayesian clustering analysis performed with STRUCTURE are shown in electronic supplementary material, figure S1.

### 3.2. Behavioural assays

All the individuals but two completed the tests. Seven out of the nine studied behavioural descriptors showed significant repeatability (table 2).

**Table 2.** Repeatability estimates of the behavioural descriptors of spontaneous activity, boldness and exploratory activity for both larvae and juveniles among different trials obtained by fitting the generalized linear mixed effects models (GLMM) implemented in the ‘rptR’ R package. *R*: repeatability (i.e. intraclass correlation coefficient); s.e.: standard error; CI: 95% confidence intervals obtained from 1000 parametric bootstrap iterations; *p*: significance, obtained by likelihood ratio tests (LRT).

	spontaneous activity in FE				boldness and exploratory activity in NE				
	larvae		juveniles		larvae			juveniles	
	mobility events	sheltering	mobility events	sheltering	latency to exit	activity %	sheltering	activity %	sheltering
*R*	0.379	0.130	0.494	0.213	0.383	0.340	0	0.318	0.224
s.e.	0.096	0.110	0.067	0.113	0.093	0.100	0.089	0.083	0.110
CI	[0.184,0.572]	[0,0.372]	[0.353,0.616]	[0,0.405]	[0.187, 0.548]	[0.124,0.526]	[0,0.297]	[0.157,0.473]	[0,0.395]
*p* [LRT]	0	0.166	0	0.045	0	0.002	0.499	0	0.031
[permutation]	0.002	0.189	0.001	0.069	0.120	0.002	1	0.002	0.019

Results from the multivariate mixed model showed significant phenotypic correlations among pairs of repeatable behaviours among individuals (table 3). We found a positive correlation between the juveniles’ spontaneous activity in the FE (mobility events) and NE (activity percentage; median correlation coefficient of 0.226) and a negative correlation between juveniles’ spontaneous activity and their sheltering behaviour in both FE and NE (median correlation coefficient −0.263 and −0.307, respectively), and with the latency to exit from the shelter of larvae in the NE (−0.257). Moreover, we found a negative correlation between the latency to exit from the shelter of larvae in the NE and their mobility events in an FE (−0.188). Finally, the latency to exit from the shelter of larvae in an NE is negatively correlated with the activity of juveniles (−0.221), and strongly correlated with the sheltering behaviour of juveniles in the NE (0.548). That is, less active individuals were more prudent and spent more time inside the shelters. These results outline consistent behavioural strategies across contexts and developmental stages.

**Table 3.** Results of bivariate Markov chain Monte Carlo (MCMC) general linear mixed effects models testing for correlations between each pair of behavioural traits measured in salamander larvae and juveniles; the best estimates of correlation coefficients (median values) are shown below the diagonal, followed by their 95% credible intervals within square brackets; correlations are considered significant (values in bold) when the credible intervals do not overlap zero.

		spontaneous activity in familiar environment (FE)			boldness and exploratory activity in novel environment (NE)			
	**trait considered**	**mobility events of larvae**	**mobility events of juveniles**	**sheltering of juveniles**	**latency to exit of larvae**	**activity of larvae**	**activity of juveniles**	**sheltering of juveniles**
FE	**mobility events of larvae**	—						
	**mobility events of juveniles**	−0.093 [−0.260;0.072]	—					
	**sheltering of juveniles**	−0.073 [−0.335;0.191]	**−0.263** **[−0.456;0.058]**	—				
NE	**latency to exit of larvae**	**−0.188** **[−0.364;0.017]**	**−0.257** **[−0.418;0.085]**	0.199 [−0.092;0.472]	—			
	**activity of larvae**	−0.050 [−0.226;0.131]	0.041 [−0.140;0.209]	−0.023 [−0.267;0.236]	0.059 [−0.088;0.196]	—		
	**activity of juveniles**	0.046 [−0.114;0.213]	**0.226** **[0.104;0.349]**	−0.044 [−0.171;0.08]	**−0.221** **[−0.370;0.071]**	0.073 [−0.088;0.251]	—	
	**sheltering of juveniles**	0.067 [−0.221;0.357]	**−0.307** **[−0.508;0.097]**	0.180 [−0.136;0.493]	**0.548** **[0.267;0.772]**	−0.090 [−0.394;0.191]	0.131 [−0.083;0.348]	—

Results from the GLMMs showed substantial behavioural differences among the individuals bearing the two alternative mitotypes (table 4). The effect of mitotype was statistically significant for all the tested behavioural traits, except for the mobility events of larvae in the FE; the latency to exit from the shelter of larvae in the NE was only marginally non-significant (*p* = 0.052).

**Table 4.** Outcomes of the generalized linear mixed models using behavioural traits as a dependent variable, the mitotype and the trials as fixed factors and the individual ID as a random factor. FE: familiar environment; NE: novel environment; s.e.: standard error; CI: confidence interval; significant *p*-values (*p* ≤ 0.05) are highlighted in bold.

variable	predictor	estimate	s.e.	CI	*p*
**spontaneous activity in FE**					
mobility events of larvae	(intercept)	20.44	2.2	16.55–25.24	<0.001
	mitochondrial lineage (south)	0.99	0.19	0.68–1.44	0.94
	trial (B)	0.8	0.03	0.75–0.85	**<0.001**
	random effects marginal *R*^2^/conditional *R*^2^	0.020/0.920			
mobility events of juveniles	(intercept)	44.36	1.59	41.23–47.50	<0.001
	mitochondrial lineage (south)	6.93	2.18	2.63–11.22	**0.002**
	trial (B)	2.48	1.31	−0.11–5.07	0.06
	random effects marginal *R*^2^/conditional *R*^2^	0.061/0.501			
sheltering of juveniles	(intercept)	1.03	0.14	0.78–1.35	0.853
	mitochondrial lineage (south)	0.33	0.07	0.22–0.50	**<0.001**
	trial (B)	0.71	0.1	0.53–0.94	**0.018**
	random effects marginal *R*^2^/conditional *R*^2^	0.186/0.417			
**exploration and boldness in NE**					
latency to exit of larvae	(intercept)	273.85	18.44	237.47–310.22	<0.001
	mitochondrial lineage (south)	−46.99	23.99	−94.32–0.35	0.052
	trial (B)	−24.82	20.48	−65.21–15.58	0.227
	random effects marginal *R*^2^/conditional *R*^2^	0.028/0.235			
activity of larvae	(intercept)	0.48	0.02	0.44–0.52	<0.001
	mitochondrial lineage (south)	0.06	0.02	0.02–0.11	**0.01**
	trial (B)	−0.05	0.02	−0.09–−0.01	**0.01**
	random effects marginal *R*^2^/conditional *R*^2^	0.068/0.356			
activity of juveniles	(intercept)	40.56	2.07	36.49–44.63	<0.001
	mitochondrial lineage (south)	6.2	2.73	0.82–11.58	**0.024**
	trial (B)	−7.44	1.99	−11.36–−3.52	**<0.001**
	random effects marginal *R*^2^/conditional *R*^2^	0.061/0.347			
sheltering of juveniles	(intercept)	0.99	0.13	0.76–1.28	0.928
	mitochondrial lineage (south)	0.67	0.12	0.47–0.94	**0.021**
	trial (B)	0.85	0.11	0.65–1.10	0.205
	random effects marginal *R*^2^/conditional *R*^2^	0.039/0.315			

**Figure 3.**
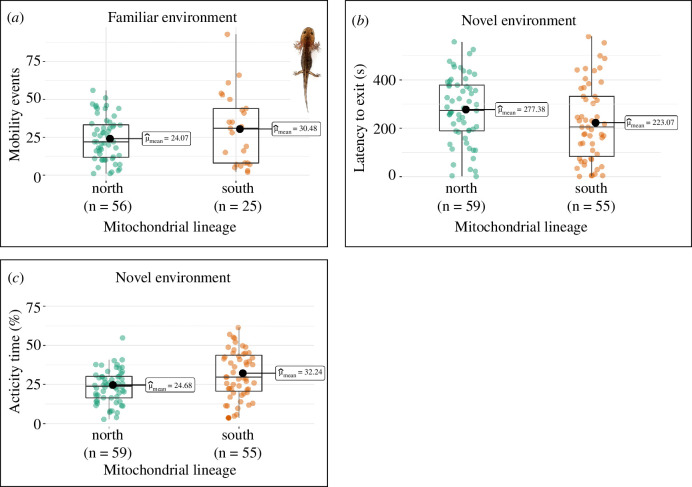
Behavioural traits assessed in fire salamander larvae in familiar and novel environments for the northern and southern mitotypes. (*a*) Spontaneous activity of larvae in FE expressed as mobility events on 18 min; (*b*) latencies to exit from the shelter in NE, in seconds; (*c*) exploratory activity of larvae in the NE expressed as the time spent in activity percentage on 10 min. Box plots show the mean (inset), the median (central line), the 25 and 75 percentile (box limits) and the lower and upper bounds (thin lines). Image obtained using the ‘ggstatsplot’ R package [90].

**Figure 4.**
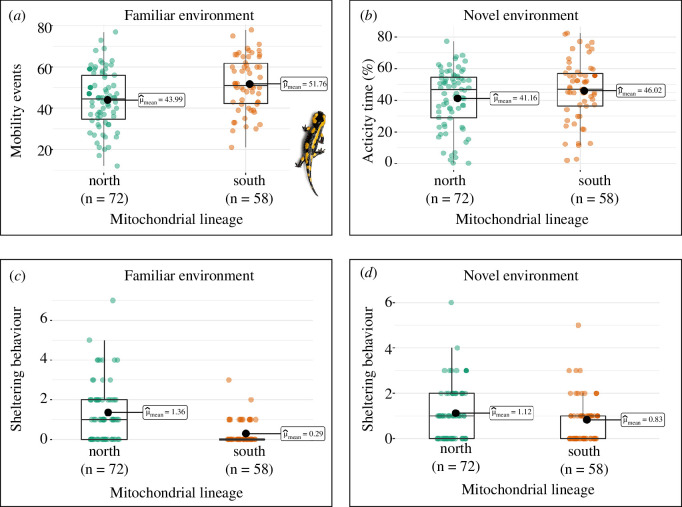
Behavioural traits assessed in fire salamander juveniles in familiar and novel environments for the northern and southern mitotypes. (*a*) Spontaneous activity of juveniles in FE expressed as mobility events on 24 h; (*b*) exploratory activity of juveniles in the NE expressed as the percentage of time spent in activity on 1 h; (*c*) sheltering behaviour of juveniles in the FE expressed as the number of times the individual comes back into the shelter; (*d*) sheltering behaviour of juveniles in the NE expressed as the number of times the individual comes back into the shelter. Box plots show the mean (inset), the median (central line), the 25 and 75 percentile (box limits) and the lower and upper bounds (thin lines). Image obtained using the ‘ggstatsplot’ R package [90].

Overall, individuals of the northern mitotype took a longer time to exit from the shelter and showed lower levels of exploratory activity in the NE than individuals of the southern mitotype. Furthermore, individuals of the northern mitotype showed lower spontaneous activity levels and higher use of the shelter in the FE than individuals of the southern mitotype (see figures 3 and 4). Interestingly, we found consistent patterns in traits tested across both the life stages: the northern mitotype showed lower spontaneous activity in the FE and lower exploratory activity in the NE, both as larvae and as juveniles.

Finally, multivariate analysis showed a significant effect of mitotype in explaining the behavioural differences across all traits together (PERMANOVA *R*^2^ = 0.06, *p* = 0.001), i.e. the northern and southern mitochondrial lineages differed in their multi-trait phenotypic variability (electronic supplementary material, figure S2).

## 4. Discussion

We found evidence that two alternative mitotypes of fire salamanders spanning the secondary contact zone are associated with two alternative behavioural profiles and that such distinctiveness is consistent across life stages. In particular, the northern mitotype was associated with a less active and less exploratory behavioural profile, both as larva and juvenile, while the southern mitotype was associated with higher boldness and a more active and exploratory behaviour. Still, such a strong behavioural differentiation is explained by the mitotype in the absence of any population genetic structure at nuclear loci, supporting a tight link between mitochondrial variation and behavioural variation in the analysed traits. In the light of the literature linking boldness-shyness, activity and exploratory behaviour to individual dispersal tendency [31–35,38–42], our data suggest the intriguing hypothesis of a behavioural underpinning for the mito-nuclear discordances observed across the secondary contact zone of *S. salamandra* in central Italy, that is, the northern mitotype could carry behavioural traits linked to dispersal and driving its rampant introgression across the secondary contact zone.

Results from this study showed substantial inter-individual variation in behavioural traits of the fire salamander. However, since biologically meaningful variability is conditioned by the consistency of individual patterns, the first important step was to demonstrate the statistical repeatability of the traits we focused on [71]. We found individual repeatability and consistency in the behaviour across life stages and contexts. Repeatability and consistency in behavioural responses over time and across contexts have been reported in many taxa, including salamanders, and have been commonly referred to instances of personalities ([91,92]; see [93] for a review). On the contrary, fewer data are available about consistency across distinct life stages ([94]; see [95] for a review). We found a substantial phenotypic correlation and consistent patterns in traits tested for both the life stages, i.e*.* the northern mitotype showed lower spontaneous activity and lower exploratory activity both as larvae and as juveniles. Consistency across distinct life stages could represent the outcome of functional links (or constraints) between physiological and behavioural traits, suggesting common underlying mechanisms affecting energy allocation and usage between the larvae and juvenile salamander stages [91,96]. It is worth noting that we found a more marked pattern in juveniles (i.e*.* higher repeatability and stronger phenotypic correlation of traits measured in juveniles, and a stronger association of these traits with the mitotype) suggesting a main role of the juvenile stage in driving the selection of dispersal-related behavioural traits. However, this pattern suggests that these personality traits could cluster together with some other life-history traits, such as metabolic activity, body size and lifespan in ‘dispersal syndromes’, as observed in many other taxa [44,97,98]. Yet, more data from physiological, morphological and performance traits [99], including data from the adult stages, are needed to corroborate this hypothesis.

We found a less active and less exploratory profile associated with the introgressed northern mitotype. This pattern is apparently in contrast with the literature reporting more active and exploratory behavioural profiles at the range edge of expanding populations [100–104]. However, growing literature has been reporting an alternative pattern, i.e. a careful and thorough exploration strategy would be favoured when expanding into NEs [49,105,106]. The pattern we found in *S. salamandra* seems to conform to this alternative scenario, whereby dispersive profiles are characterized by long decision-making times in facing the risks of an unfamiliar situation. The higher use of the shelter and the lower exploratory activity in the unfamiliar environment shown by the northern mitotype, both in larvae and juveniles, outline a prudent strategy reflecting a reactive coping style [107]. According to the coping style theory, reactive individuals are ‘slow-thorough’ explorers (*sensu* [107]) as they tend to rely more on the information on current environmental conditions, which may take time to acquire, resulting in a cautious but accurate strategy of exploration [45,108]. This strategy could favour the establishment of a good feeding ground, due to a more efficient energy balance, and the capacity to perform prolonged efforts [109–111], resulting in higher fitness in stressful situations such as facing newly colonized environments [111,112]. Interestingly, better performances for a dispersal profile characterized by a less active but more cautious and accurate strategy of exploration seem to fit well with a species characterized by marked territoriality and strong breeding site fidelity through most of its life [57–59]. Thus, the ‘slow-thorough’ profile associated with the fire salamander northern mitotype might have favoured this mitotype introgression because of a higher fitness in recently expanded populations [113–115]. It is worth noting that mitochondria are important modulators of the physiological stress response [116], whose individual differentiation is a central tenet of the coping style theory. The pattern we found in the two salamander lineages reflecting behavioural profiles similar to the proactive and reactive styles could lead to the hypothesis that mitochondrial function is a major determinant of the different coping styles that could therefore depend upon the type of mitochondrial genome an individual carries. Interestingly, our results suggest some context dependency of the mitochondrial influence on behaviour, i.e. the mitotype effect was more pronounced on juveniles compared with larvae, and more in NE with respect to FE. Future research should specifically target mitochondria to better understand the roots of personality differences and stress coping.

Our results support the hypothesis that specific mitotypes could carry specific, potentially adaptive life-history traits linked to the individual movement tendency and that such a link could lead to mitochondrial genome introgression across a secondary contact zone, irrespective of the nuclear genome background. In this respect, it is worth acknowledging that we assessed genetic structure by employing a limited number of microsatellite loci, which should not be deemed fully representative of the differentiation at the level of the entire nuclear genome. Indeed, our study does not allow us to disentangle if the behavioural differences are caused by the mitotype *per se*, or are an effect of mito-nuclear combinations. We expect that genome-scale investigations (see [117]) could reveal areas of the genome differentiated between the two mitotypes/behavioural profiles, as well as patterns of co-introgression, especially among nuclear genes tightly linked to the mitochondrial metabolism.

The key role of behavioural polymorphisms in explaining biogeographic patterns is being increasingly recognized, with the hypothesis that divergent evolution of personalities, due to different selection pressures across the expansion range, could lead to a non-random distribution of multiple dispersal strategies—and associated genotypes—among populations (see [50,51] and references therein). However, from the first indication that individuals characterized by fast-bold behavioural profiles are the main contributors to a successful range expansion, more complex scenarios are emerging. By suggesting a role for interindividual behavioural variation—and for slow-thorough profiles in particular—in the formation of mito-nuclear discrepancies along secondary contact zones, our results support such emerging complexity in the formation of biogeographic patterns.

## Data Availability

The datasets generated and/or analysed during the current study are available in the Zenodo repository at the following link [118]. The link also includes the video recordings of the four behavioural tests performed on the individual 203; because of size limitations, the videos of the test performed on all the other individuals are stored in a private server and available upon request from the corresponding author. Supplementary material is available online [119].
